# Bullying History and Mental Health In University Students: The Mediator Roles of Social Support, Personal Resilience, and Self-Efficacy

**DOI:** 10.3389/fpsyt.2019.00960

**Published:** 2020-01-14

**Authors:** Muyu Lin, Dieter Wolke, Silvia Schneider, Jürgen Margraf

**Affiliations:** ^1^ Department of Clinical Psychology and Psychotherapy, Mental Health Research & Treatment Center, Ruhr-Universität Bochum, Bochum, Germany; ^2^ Department of Psychology and Warwick Medical School, University of Warwick, Coventry, United Kingdom; ^3^ Department of Clinical Child and Adolescent Psychology of the Faculty of Psychology, Mental Health Research & Treatment Center, Ruhr-Universität Bochum, Bochum, Germany

**Keywords:** bullying, perpetrators, social support, self-efficacy, resilience, cross-cultural differences, positive mental health, mental illness

## Abstract

Bullying victimization by peers is highly prevalent in childhood and adolescence. There is convincing evidence that victimization is associated with adverse mental health consequences. In contrast, it has been found that perpetrators suffer no adverse mental health consequences. These findings originate from Western countries such as Germany but have rarely been investigated in collectivistic societies such as China. Furthermore, it has been rarely studied whether positive intrapersonal characteristics (e.g., personal resilience and self-efficacy) and interpersonal positive resources (e.g., social support) may mediate the impact of bullying on mental health. The current study used a path analytic model to examine, firstly, whether previous bullying experiences (both victimization and perpetration) are associated with current positive and negative mental health in university students and, secondly, whether these influences are mediated by social support, resilience, and self-efficacy. The model was tested in 5,912 Chinese and 1,935 German university students. It was found that in both countries, higher victimization frequency was associated with lower levels of social support, personal resilience, and self-efficacy, which in turn predicted poorer mental health. Moreover, and only in China, perpetration was negatively associated with social support and personal resilience but not self-efficacy. In contrast, in the German sample, perpetration experience was found to enhance one's self-efficacy, and the later was associated with better mental health. The results support a mediation model in which social support, personal resilience, and self-efficacy partially mediate the influence of victimization on mental health in both countries. For the relationship between perpetration and mental health, self-efficacy was the only full mediator in Germany, whereas in China, both social support and personal resilience were partial mediators. In conclusion, peer victimization has adverse effects on mental health in both Germany and China. Only in China, however, is perpetration also associated with adverse mental health outcomes. In contrast, getting ahead by bullying in an individualistic society such as Germany is associated with increased self-efficacy and mental health. The differences found between an individualistic country and a collectivistic country have important implications for understanding and planning interventions to reduce bullying.

## Introduction

Peer bullying at school is highly prevalent and has become an international concern (e.g., 1, 2). Victimization has been universally found to be associated with cross-sectional and long-term adverse mental health consequences, including more severe depression and anxiety symptoms (e.g., 3–5) and lower levels of positive mental health (e.g., 4).

In contrast, the relationships between bullying perpetration and health problems are not consistent across countries (2). In some countries such as Germany, Austria, the UK, the USA, and Denmark, bullies appear to be as healthy as non-involved peers, in terms of adult mental and general health (5, 6), except for a higher risk for antisocial personality (7) and alcohol use (2). However, in other countries such as China, Greece, or Israel, perpetrators have reported worse health problems and emotional adjustment (2, 8). Furthermore, bullies may perceive less social support than non-involved students in the USA and China (8, 9). The differences between bullies in different countries indicate that the same behavior may have different consequences depending on context and societal norms. Thus, a cross-national study that applies the same measures in different cultures may help to clarify the relationship between perpetration and mental health.

Only recently has research focused on factors that may help to explain how being bullied may be associated with adverse mental health outcomes (e.g., 10, 11). An increasing amount of urecharacteristics (e.g., personal resilience and self-efficacy) can promote mental well-being (12–14). These may be protective factors that mitigate the negative impact of bullying experience on mental health, meanwhile, they may also be influenced by the bullying experiences.

As one of the most prominent protective factors, perceived social support plays an essential part in preventing mental illness (e.g., 12, 13, 15). It has a remarkably consistent positive association with positive mental health (e.g., 16, 17). Perceived social support refers to an individual's feeling or evaluation of whether the social network is supportive enough to facilitate the individual's coping with tasks and stress or to achieve personal goals (18, 19). The link between social support and bullying has been well established, with poor social support highly associated with victimization by peers (e.g. 20, 21). Stress may erode the perception or effectiveness of social support (22). For instance, longitudinal evidence has shown that “continuous victims of bullying” had worse school attendance rates, which further isolated them from peers and undermined a healthy peer relationship (23). Furthermore, social support has been shown to mediate the negative effect of workplace or school bullying on positive or negative well-being (24, 25).

While some use friendships and family as protective buffers, others may rely on their resilience to overcome the adversity of victimization (10). Resilience can manifest in several ways. Personal resilience refers to the capacity to adapt, recover, and avoid potential deleterious effects after facing overwhelming adversity (14). Children and adolescents are in a constant process of development. Thus, their resilience trait is more likely to be influenced by situational factors such as bullying involvement during primary or secondary school periods. For example, negative life events negatively predict resilience in students (26) and parental HIV longitudinally affected resilience in children (27). Indeed, research has shown that resilience trait mediates the relationships between workspace bullying and physical strain (28) and between primary school bullying and depressive symptoms (29).

Another essential positive factors in stress regulation is self-efficacy. The perception of self-efficacy is the belief that one can perform novel or challenging tasks and attain desired outcomes, indicating a self-confident view of one's own capability to deal with stressors in life [see Social Cognitive Theory, (30, 31)]. High self-efficacy is associated with higher levels of optimism and life satisfaction (28, 33) and lower anxiety and depression (34). Meanwhile, prior experience is one of the most influential factors that shape self-efficacy (35). It is likely that a negative peer experience (i.e., victimization) or a mastery experience (i.e., perpetration) influence one's self-efficacy appraisal. For instance, previous research indicates that self-efficacy mediates the effect of stressful life events or daily stressors on both positive and negative mental health in samples from different cultures (36, 37).

Unlike social support and personal resilience, results on the relationship between self-efficacy and bullying involvement are mixed. In some research, both victimization and perpetration were found to be negatively associated with overall self-efficacy [Greek elementary school children: 38; Turkish middle school students: (39)]. In some cases, it has been found that victims have lower self-efficacy than bullies and those not involved in Chinese primary and German secondary school bullying. Bullies, on the other hand, do not tend to differ from not-involved peers in self-efficacy (8). There are also studies indicating that firmer self-efficacy beliefs are positively correlated to high levels of self-reported cyberbullying behaviors (40). A possible explanation for the mixed results regarding self-efficacy may be that a substantial number of persons are involved in both bullying perpetration and victimization (i.e., so-called bully-victims). Therefore, in the current study, the correlations between perpetration and victimization were controlled.

**Figure 1:**
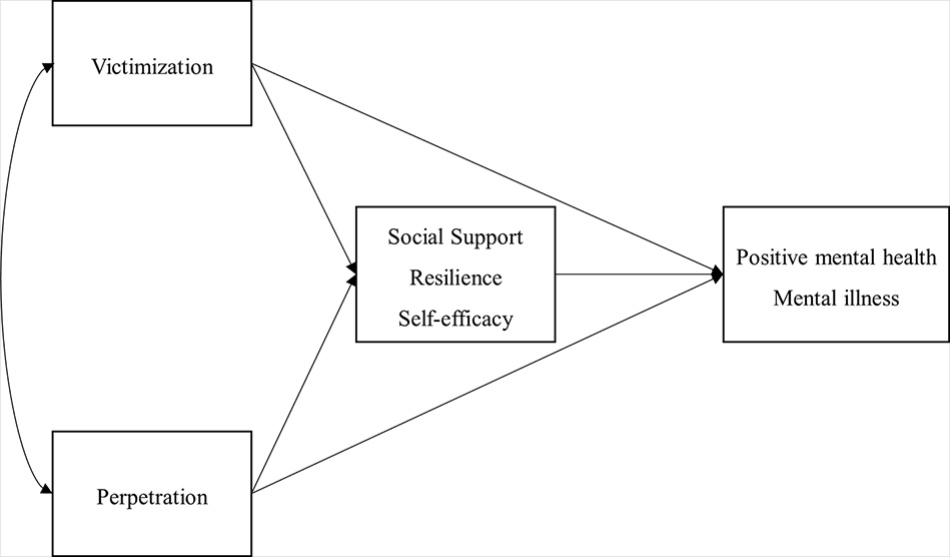
A hypothesized mediation model for bullying and mental health.

In sum, there is some consistency in the findings when it comes to social support and personal resilience as single mediators in the relationship between victimization and mental health. The role of self-efficacy has not yet been established. Thus social support, personal resilience, and self-efficacy may be considered potential factors that protect against being bullied and may explain the impact of previous bullying severity on mental health. Therefore, the current study aimed to explore the role of perceived social support, personal resilience, and self-efficacy in the relationship between *previous* peer bullying experience (both victimization and perpetration) and *current* mental health (both positive mental health and mental illness symptoms) in university students using a mediation model (see **Figure 1** for a hypothesized model). Bullying experience was measured with a retrospective inventory regarding victimization and perpetration frequency from primary schools to current universities. Our work aims to add insight into the relationship between school bullying and its long-term consequences during university. Both perpetration and victimization experiences were examined in one model in order to control for the correlation between them. Adding perpetration into the model was also predicted to expand our knowledge of how bullying behaviors impact one's mental health. Moreover, in order to expand on previous works that typically focused on only the mental illness, both the positive and negative aspects of mental health were outcome variables [measured by the Positive Mental Health scale, PMH; (41); and the Depression, Anxiety, and Stress Scale, DASS; (42)].

Furthermore, as reviewed above, there appear to be cultural differences in the effects of bullying perpetration on well-being and mental health. So far, our knowledge of bullying consequences is primarily based on studies carried out in western, individualistic societies. In more collectivistic cultures such as China, however, bullying and its mechanisms have rarely been investigated. There is evidence that bullies in China also suffer from concurrent or long-term problems such as poor life satisfaction, depression, suicide ideation, or psychoticism (e.g., 8, 43, 44), unlike the phenomena found in western countries where bullies typically do well (2, 5, 6). Therefore, the hypothesized model was tested within two separate samples: university students in China, a country that fosters Eastern Asian group-oriented culture (e.g., 45, 46); and students in Germany, a West European individualistic country, where the ties between individuals are relatively loose (45).

Based on the research regarding bullying and its aversive consequences on mental health and the protective role of social support, personal resilience, and self-efficacy (e.g., 3, 4, 10, 12, 32), it is hypothesized that in both countries, (a) social support, personal resilience, and self-efficacy would be positively related to PMH and negatively related to DASS; (b) victimization experience would be positively related to DASS and negatively related to PMH and (c) social support, personal resilience, and self-efficacy would mediate the relationship between victimization and mental health. Giving that bullies reported different mental health levels across various countries (2, 5, 8), we further hypothesized cross-cultural differences regarding the paths on perpetration.

## Method

### Participants

This study is part of the Bochum Optimism and Mental Health (BOOM) research project, which is a large-scale cross-cultural longitudinal investigation in mental health. The Ethics Committee of the Faculty of Psychology at Ruhr University Bochum approved the project. Chinese data were collected either by paper-pencil survey or online questionnaires, while German data were all collected via an online survey.

In total, 5,912 Chinese students from Capital Normal University (Beijing city), Shanghai Normal University (Shanghai city), Nanjing University (Nanjing city), Hebei United University (Tangshan city), and Guizhou University of Finance and Economics (Guiyang city) participated in the 2015 survey. All participants were in the fourth year of bachelor degree studies (age: 21.54 ± 1.20). Among them, 3,301 (60.0%) were female and 2,202 (40.0%) were male; 3,403 (60.1%) came from low affluent families, 1,687 (29.8%) from medium affluent families, and 573 (10.1%) from high affluent families. Family affluence was measured and classified based on the scores on the 4-item Family Affluence Scale-II (47).

The German sample consists of 1,935 students (age: 21.73 ± 4.93) of Ruhr University Bochum (Bochum city) who took the survey at least once between 2015 and 2017. Among them, 1166 (61.7%) were female while 725 (38.3%) were male; 242 (15.7%) came from low affluent families, 812 (52.5%) from medium, and 492 (31.8%) from high affluent families; 1156 were in the freshman year, 105 in the sophomore year, 53 in the junior year, 99 in the senior year, 352 in the fifth year or higher, and 68 were in Ph.D. programs.

### Questionnaires

#### Bullying History

Peer victimization and perpetration experiences at primary school, secondary school, and currently at university were collected with the Retrospective Bullying Questionnaire [modified from (48)]. Behaviors of direct, relational and cyberbullying were first described. Participants rated how frequently they perpetrated or received (victimization) the described behavior during each school period (primary school, secondary school, current university) from 1 (*never*), 2 (*once or twice*), 3 (*occasionally*), 4 (*about once a week*), to 5 (*several times a week*). The three victimization questions across all periods were summed for a total victimization score, while the three perpetration questions were summed for a total perpetration score. The Retrospective Bullying Questionnaire was test-retested in 287 German students with a one-year gap. The one-year test-retest reliability was.81 for school victimization and ranged from.55 to.60 for school perpetration.

#### Depression, Anxiety, and Stress Scale (DASS)

The 21-item DASS (42) assesses depression, anxiety, and stress symptoms (seven items for each) from the last seven days. Participants checked agreement on a four-point Likert scale from 0 (*did not apply to me at all*) to 3 (*applied to me very much or most of the time*). A higher score indicates severer mental illness symptoms. Cronbach's alpha was.93 in the German sample and.96 in the Chinese sample.

#### Positive Mental Health Scale (PMH)

The 9-item PMH (41) measures positive aspects of emotional well-being and health on 4-point Likert scales ranging from 0 (*do not agree*) to 3 (*agree*). A higher score indicates better general positive mental health. Cronbach's alpha was.91 in the German sample and.96 in the Chinese sample.

#### Resilience Scale

The 11-item Resilience Scale (49) is a short unidimensional version of the 25-item Resilience Scale from (14), which measures psychosocial stress-resistance (e.g., personal competence and acceptance of self and life) on scales ranging from 1 (disagree) to 7 (agree). Higher scores indicate a higher level of resilience. Internal consistency was.87 in the German sample and.90 in the Chinese sample.

#### Brief Perceived Social Support Questionnaire (F-SozU K-6)

The 6-item F-SozU (50) assesses general support that one perceives from the social network. Participants indicated agreement on 5-point Likert scales ranging from 1 (*not true at all*) to 5 (*very true*). Higher scores indicate a higher level of perceived social support. Cronbach's alpha was.87 in the German sample and.90 in the Chinese sample.

#### General Self-Efficacy Scale (GSE)

The 10-item GSE (51) was used to assess a general sense of one's ability to cope when facing unexpected situations. Items are rated on a 4-point likely scale ranging from 1 (*not agree*) to 4 (*totally agree*). Higher sum scores indicate a greater sense of self-efficacy. In the German sample, Cronbach's alpha was.88, and in the Chinese sample, .93.

### Data Analysis

Multivariate analysis of variance (MANOVA) was used to examine the difference in bullying frequency (victimization and perpetration) at each school period between China and Germany. In order to define the relationship between bullying experience, positive factors, and mental well-being, Mplus [version 7.4, (52)] was used to test the path analytic model. Full information maximum likelihood (FIML) estimation was used. The hypothesized model was defined with two correlated predictors (victimization and perpetration), three inter-correlated mediators (social support, personal resilience, and self-efficacy), and two correlated dependent variables (DASS and PMH). Sum scores of all the scales were entered into the model. Bias-corrected bootstrapping (5000 times) was applied for testing the significance of indirect effects (53). Then, insignificant paths were removed one by one to simplify the model. Final models contained only significant paths. An adequate model fit was determined by a nonsignificant chi-square statistic, a root mean square error of approximation (RMSEA) <.06, a comparative fit index (CFI) >.95, and a standardized root-mean-square residual (SRMR) <.08 (54). The effect size of the standardized regression coefficient was interpreted as small (.14), medium (.39), and large (.59) based on Cohen (55); while the effect size of standardized indirect effects was interpreted as small (.01), medium (.09), and large (.25) as suggested by Kenny and Judd (56). The datasets for this study can be found in the online **Supplementary Material**.

## Results

### Bullying Frequency in Both Countries

**Table 1** presents the self-reported bullying frequency at primary, secondary school, and university. Results from MANOVA showed that both countries differed significantly for all periods; however, the effect size of bullying at university was trivial (η^2^_part._ <.01). German students reported more frequently being bullied and bullying others than Chinese students during primary and secondary school.

**Table 1:** Means (M) and standardized deviations (SD) of bullying frequency in each school period.

Bullying	Variables	China			Germany			*F* (1, 7728)	η^2^_part._
		*M*	*SD*	*N*	*M*	*SD*	*N*		
Victimization	Primary school	1.42	0.82	5910	2.12	1.18	1935	859.50***	.100
	Secondary school	1.22	0.61	5892	2.30	1.27	1935	2533.05***	.247
	University	1.11	0.46	5890	1.21	0.61	1934	52.71***	.007
Perpetration	Primary school	1.18	0.58	5852	1.45	0.70	1935	288.55***	.036
	Secondary school	1.12	0.49	5858	1.51	0.74	1934	711.74***	.084
	University	1.08	0.41	5884	1.06	0.28	1933	7.86**	.001

***: *p* <.001; **: *p* <.01.

### Correlation Table

**Table 2** presents the correlations between the variables. All variables were found to be significantly correlated with each other (*p* <.05), except for perpetration, which was not correlated with personal resilience and self-efficacy in the German sample. As expected, in both countries, victimization was positively related to perpetration and DASS, and negatively related to social support, personal resilience, self-efficacy, and PMH. Moreover, the three positive factors were positively inter-correlated with each other and with the two outcome measures. Additionally, in China, the effect sizes between perpetration and other variables were small to modest, whereas the same correlation in Germany had only trivial to small effects.

**Table 2:** Means (M) and standardized deviations (SD) of measures and correlation table.

Variables	*M*	*SD*	*N*	Victimization	Perpetration	Social support	Resilience	Self-efficacy	PMH
China									
Victimization	3.74	1.46	5,912	1					
Perpetraion	3.36	1.23	5,903	.465**	1				
Social support	24.45	4.20	5,902	−.189**	−.142**	1			
Resilience	59.17	9.33	5,885	−.151**	−.118**	.553**	1		
Self-efficacy	29.41	5.00	5,904	−.161**	−.072**	.472**	.589**	1	
PMH	20.47	4.95	5,906	−.212**	−.143**	.539**	.572**	.616**	1
DASS	8.48	10.75	5,896	.293**	.244**	−.349**	−.330**	−.248**	−.443**
Germany									
Victimization	5.63	2.34	1,935	1					
Perpertration	4.02	1.35	1,935	.262**	1				
Social support	25.38	4.61	1,889	−.253**	−.064**	1			
Resilience	58.28	9.79	1,889	−.173**	−.026	.523**	1		
Self-efficacy	28.54	4.98	1,888	−.180**	.044	.451**	.706**	1	
PMH	17.85	5.91	1,887	−.279**	−.052*	.561**	.674**	.666**	1
DASS	16.55	12.31	1,885	.276**	.057*	−.434**	−.520**	−.530**	−.708**

### Mediated Path Analytic Model Within the German Sample

**Figure 2:**
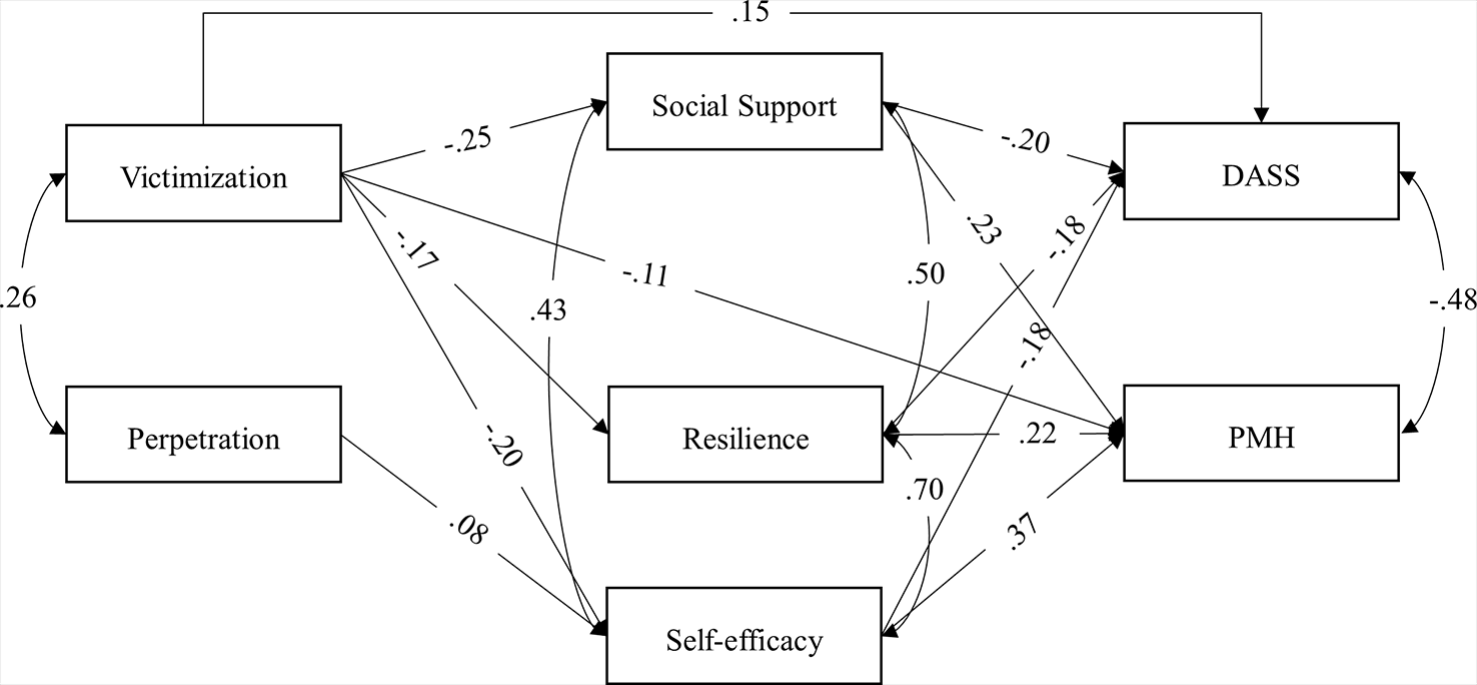
Final path mediated model for the effects of bullying, social support, personal resilience, and self-efficacy on positive and negative well-being in the German sample. Regression paths (single-arrow) and correlation paths (curved double-arrow) were all significant on at least.05 level. Standardized coefficients are shown. DASS, Depression, Anxiety, and Stress Scale. PMH, Positive Mental Health Scale.

The results of the final mediated path model in the German sample indicate an excellent fit of the data, RMSEA <.0001 (90% confidence interval from <.0001 to.027), CFI = 1, SRMR =.004. The standardized path coefficients (*p* <.001) of the final model are shown in **Figure 2**. Victimization experience was negatively linked with all three mediators and the two dependent variables, and the three mediators further associated negatively with DASS and positively with PMH, suggesting that social support, personal resilience, and self-efficacy partially mediated the effect of victimization on the two mental health measures. Perpetration experience was significantly linked only with self-efficacy, the later further regressed positively on PMH and negatively on DASS, suggesting that self-efficacy fully mediated the effect of perpetration on mental health. The correlations between the two predictors, the three mediators, and the two dependent variables were all significant at.001 level. The effect sizes of the direct and indirect effects from the bootstrapping are presented in **Table 3**. In addition, the final model explained 58.1% of the variance in PMH, 37.0% in DASS, 3.0% in personal resilience, 3.9% in self-efficacy, and 6.4% in social support.

**Table 3:** Standardized total indirect, specific indirect, and direct effects and their 95% confidence intervals (C.I.).

Predictor	Dependent variable	Total indirect effect [95% C.I.]	Specific indirect effect			Direct effect [95% C.I.]
			Social support [95% C.I.]	Resilience [95% C.I.]	Self-efficacy [95% C.I.]
China						
Victimization	PMH	−.12 [−.14, −.10]	−.04 [−.05, −.03]	−.03 [−.03, −.02]	−.06 [−.07, −.05]	−.06 [−.09, −.04]
Victimization	DASS	.05 [.04,.06]	.03 [.02,.04]	.02 [.02,.03]	/	.18 [.14,.21]
Perpetration	PMH	−.03 [−.04, −.02]	−.02 [−.02, −.02]	−.01 [−.02, −.01]	/	−.03 [−.05, −.01]
Perpetration	DASS	.03 [.02,.04]	.01 [.01,.02]	.01 [.01,.02]	/	.11 [.08,.14]
Germany						
Victimization	PMH	−.18 [−.21, −.14]	−.06 [−.07, −.04]	−.05 [−.07, −.04]	−.07 [−.08, −.05]	−.11 [−.14, −.08]
Victimization	DASS	.13 [.11,.16]	.04 [.03,.06]	.04 [.02,.05]	.06 [.04,.07]	.15 [.12,.18]
Perpetration	PMH	.03 [.02,.04]	/	/	.03 [.02,.04]	/
Perpetration	DASS	−.02 [−.04, −.01]	/	/	−.02 [−.04, −.01]	/

### Mediated Path Analytic Model in the Chinese Sample

**Figure 3:**
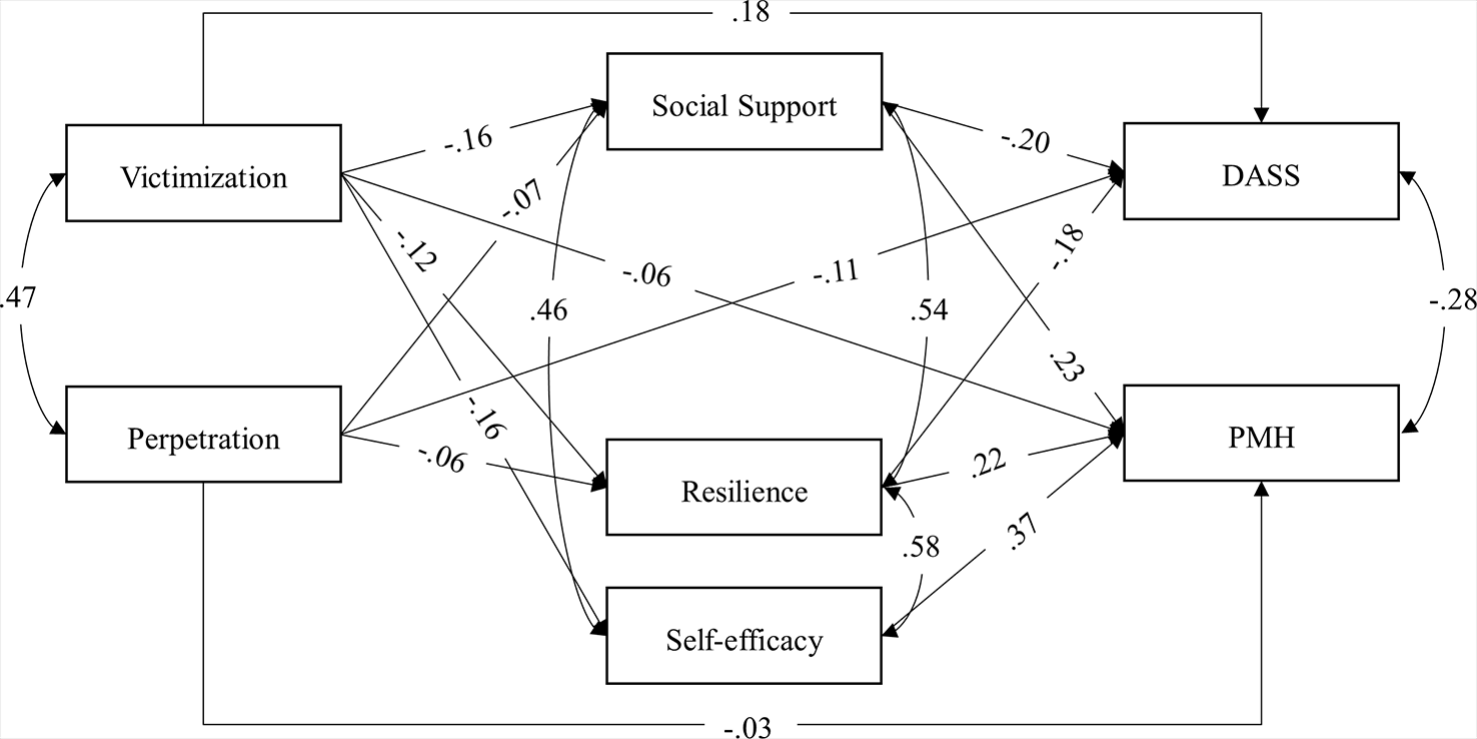
Final path mediated model for the effects of bullying, social support, personal resilience, and self-efficacy on positive and negative well-being in the Chinese sample. Regression paths (single-arrow) and correlation paths (curved double-arrow) were all significant on at least.05 level. Standardized coefficients are shown. DASS, Depression, Anxiety, and Stress Scale. PMH, Positive Mental Health Scale.

The results of the final mediated path model in the Chinese sample also indicate an excellent fit of the data, RMSEA <.0001 (90% confidence interval from <.0001 to.024), CFI = 1, SRMR =.002. The standardized path coefficients are shown in **Figure 3**. Victimization experience was negatively linked with all three mediators and the two dependent variables, while perpetration frequency was negatively linked with personal resilience and social support and the two dependent variables but not with self-efficacy. All three positive factors were positively associated with PMH, while only social support and personal resilience further regressed on DASS. The results indicate that social support, personal resilience, and self-efficacy partially mediated the effect of victimization on mental health and that only social support and personal resilience partially mediated the effect of perpetration on mental health. The direct and indirect effects of the mediation are presented in **Table 3**. Moreover, all the correlations were significant at.001 level. In addition, the final model explained 49.0% of the variance in PMH, 20.8% in DASS, 2.6% in personal resilience and self-efficacy, and 3.9% in social support.

## Discussion

The main aim of this study was to test the mediators of previous bullying experience regarding the outcomes of both positive and negative mental health in university students in China and Germany. For both countries, it was found that social support, personal resilience, and self-efficacy partially mediate the effect of previous victimization experience on current well-being and mental illness. In contrast, cultural differences were observed for the relationship between perpetration and positive and mental health. For Germany, only self-efficacy fully mediated the effect of perpetration on mental health: more frequent perpetration promoted higher mental health status via a higher level of self-efficacy. Conversely, for students in China, social support and partially resilience partially mediated the effect of perpetration on mental health. More specifically, more frequent bullying perpetration was linked with a lower level of social support perception and lower personal resilience, which in turn was found to be associated with worse mental health.

In both countries, social support, personal resilience, and self-efficacy partially mediated the negative effect of victimization on mental health, with medium-sized total indirect effects. The results replicate previous findings on similar social resources and positive traits (e.g., 24, 28, 29, 38, 57) and indicate that the long-term adverse emotional consequences of being bullied are partly explained by less social support, lower personal resilience and lower self-efficacy levels. The current results further provide some initial evidence of an important role for self-efficacy, which revealed the strongest indirect mediating effect in our data. Bullying interventions may consider promoting the social resources and the self-efficacy of the victims in order to reduce the negative impact of victimization. However, there was also a direct effect of bullying victimization, indicating that even if social support, personal resilience or self-efficacy is high, a negative effect of being excluded and beaten may not be avoided.

The relationships between perpetration, positive factors, and mental well-being were different across countries. In China, bullying others more frequently, like being bullied, was associated with a lower level of personal resilience and support perception; whereas in Germany, bullying others was unrelated to the level of social support or personal resilience, but instead even weakly increased one's self-efficacy. The results indicate that bullies from two different cultures, Germany and China, face different psychological consequences of their perpetration behavior. The associations of perpetration with positive factors were different as well. Those involved in bullying in China were less personally resilient and socially supported and had more severe mental illness symptoms (8). Thus, providing social support and strengthening personal resilience may reduce bullying perpetration in China. In contrast, in Germany, bullies were as socially supported and personally resilient but even more self-efficient than those not involved in any bullying. This is consistent with previous findings that bullying is little socially sanctioned and conducted by students who are competent social manipulators with good emotional well-being (e.g., 5, 6, 58, 59).

Cultural differences were also found in the relationship between positive and negative mental health. For instance, the effect size of the correlation between PMH and DASS was smaller in China than that in Germany. Moreover, self-efficacy had a stronger association, as indicated by the path coefficient in **Figure 3**, with PMH than with DASS in Germany. This phenomenon is more pronounced in the China sample, where self-efficacy had a significant association with PMH but not with DASS. On the one hand, these results are in line with Karademas (60), who proposed that the buffering effect of self-efficacy is greater for positive than for negative mental health. On the other hand, it may be that self-efficacy may not be related to depression or anxiety in China. In China, many people believe that uncontrollable or unexpected events or “fate” (*Tianming*) may sometimes impact the outcome of ones' best endeavors. Thus, those having high self-efficacy may face greater disappointment, while having low self-efficacy may also link to a greater sense of powerlessness. In Germany, in contrast, having higher self-efficacy not only promoted PMH but also prevented mental illness at a certain level. Taken together, it appears that the difference between the latent constructs measured by PMH and DASS was greater in China than in Germany.

While the large sample size, cross-cultural design (allowing for direct comparison of bullying involvement in Germany and China), and the inclusion of mediators are major strengths of the current study, there are also limitations. The measure of bullying history was retrospective and self-reported. However, test–retest showed high reliability over one year. Nevertheless, reported associations need to be interpreted cautiously and require replication in prospective studies. The large sample size did allow us to detect small effects. Thus, when interpreting our results, not only the significance of paths but also the effect sizes should be considered, especially regarding the effects between perpetration and other variables (56). In addition, the current study chose three representative positive factors as a start of the coping/recourse model of bullying; however, there may be more critical mediators, especially for perpetration, that were not tested in our study. Further studies may consider other protective or buffering factors and expand the model upon the three mediators examined in the current study.

In sum, the current study found that social support, personal resilience, and self-efficacy play essential roles in regulating the influences of victimization on later mental well-being across countries considered as individualistic or collectivistic. Thus strengthening social support, personal resilience and self-efficacy are likely to help to mitigate the ill effects of peer victimization. In contrast, mechanisms of how bullying perpetration associates with mental health differ between individualistic and collectivistic cultures. In Germany, bullying increases self-efficacy and has even small positive effects on mental well-being. In contrast, in a collectivistic society such as China, bullying others is associated with reduced social support and decreased personal resilience and negative mental health. Bullying may be seen as breaking the social norms of caring for others. The model proposed here needs to be explored longitudinally and applied to the development of strategies that build psychological personal resilience and resource in bullying victims.

## Data Availability Statement

All datasets generated for this study are included in the article/**Supplementary Material**.

## Ethics Statement

This study is part of the Bochum Optimism and Mental Health (BOOM) research project, which is a large-scale cross-cultural longitudinal investigation in mental health. The project was approved by the Ethics Committee of the Faculty of Psychology at Ruhr University Bochum.

## Author Contributions

All authors listed have made a substantial, direct and intellectual contribution to the work, and approved it for publication.

## Funding

This study was supported by Alexander von Humboldt Professorship awarded to the last author by the Alexander von Humboldt-Foundation.

## Conflict of Interest

The authors declare that the research was conducted in the absence of any commercial or financial relationships that could be construed as a potential conflict of interest.
